# HO-1 inhibits migration of leukemic cells

**DOI:** 10.18632/oncotarget.21044

**Published:** 2017-09-19

**Authors:** Mariusz Z. Ratajczak

**Affiliations:** Stem Cell Institute at James Graham Brown Cancer Center, University of Louisville, KY, USA and Department of Regenerative Medicine Warsaw Medical University, Warsaw, Poland

**Keywords:** complement cascade, heme oxygenase 1, p38 MAPK inhibitors, HO-1 activators, leukemia

The complement cascade (ComC) is a crucial element of innate immunity and is involved in several processes related to fighting infection by releasing active mediators, including C3a and desArgC3a, which result from cleavage of complement component 3 (C3), and C5a and desArgC5a, which result from cleavage of complement component 5 (C5) [1-3]. In parallel, evidence has accumulated that, in addition to their immunological functions, ComC cleavage fragments modulate stem cell migration during organogenesis [2]. The ComC also orchestrates the egress of normal hematopoietic stem/progenitor cells (HSPCs) from bone marrow (BM) into peripheral blood (PB) in a process known as mobilization and mediates homing and engraftment of HSPCs in hematopoietic organs after transplantation [3]. Interestingly, these pro-mobilization and pro-homing effects are indirect, as they involve other cells in the hematopoietic microenvironment. For example, although HSPCs express the functional C5a receptor (C5aR, also known as CD88), and respond to stimulation by C3a, surprisingly these cells do not show spontaneous chemotaxis in response to C3a, C5a, desArgC3a, or desArgC5a [3]. In contrast, all these C3 and C5 cleavage fragments promote migration of already differentiated hematopoietic cells, including granulocytes, monocytes, lymphocytes, and NK cells [1-3].

On the other hand, the ComC plays a role in the pathogenesis of several solid tumors by modifying tumor cell growth, while affecting metastatic potential and the response to therapeutics [2, 4-7]. Nevertheless, in contrast to solid tumors, the potential involvement of the ComC in leukemia has not been studied extensively. To fill in this knowledge gap, we asked whether human leukemia cell lines and primary patient leukemic blasts express functional C3 and C5 cleavage-fragment receptors (C3aR and C5aR, respectively) and whether activation of the ComC and release of C3a and C5a anaphylatoxins affects the biology of leukemic cells [1]. This question was prompted by our hypothesis that infections accompanying leukemia and/or the application of chemotherapy in leukemic patients trigger activation of the ComC and the release of potent mediators derived from C3 and C5 cleavage. In our experiments we employed several established human myeloid and lymphoma cell lines, purified CD33^+^ blasts from leukemia patients, and studied the effect of C3a, C5a, desArgC3a, and desArgC5a on the proliferation, survival, migration, and adhesion of these cells [1].

Interestingly, we found that human leukemia cell lines as well as clonogenic blasts from CML and AML patients express C3a- and C5a-binding receptors at the mRNA (RT-PCR) and protein (FACS) levels, and these receptors respond to C3a and C5a stimulation by phosphorylation of p42/44 and p38 MAPK and AKT. We also observed that, while C3 and C5 cleavage fragments did not stimulate proliferation of leukemic cells, they induced random motility (chemokinesis) of these cells and increased their adhesion [1]. This random motility of cells is based on cell migration in all directions, in contrast to chemotaxis, which is a unidirectional movement of cells up a chemoattractant gradient. Like chemotaxis, chemokinesis leads to egress of cells from their primary locations and to the spread of migrating cells in the tissues. Since, as mentioned above, the ComC may be activated in leukemic patients by coexisting infections or in response to chemotherapy, C3 and C5 cleavage fragments may facilitate the unwanted spread of leukemic cells in the hematopoietic organs as well as their egress into PB, as we demonstrated [1].

**Figure 1:**
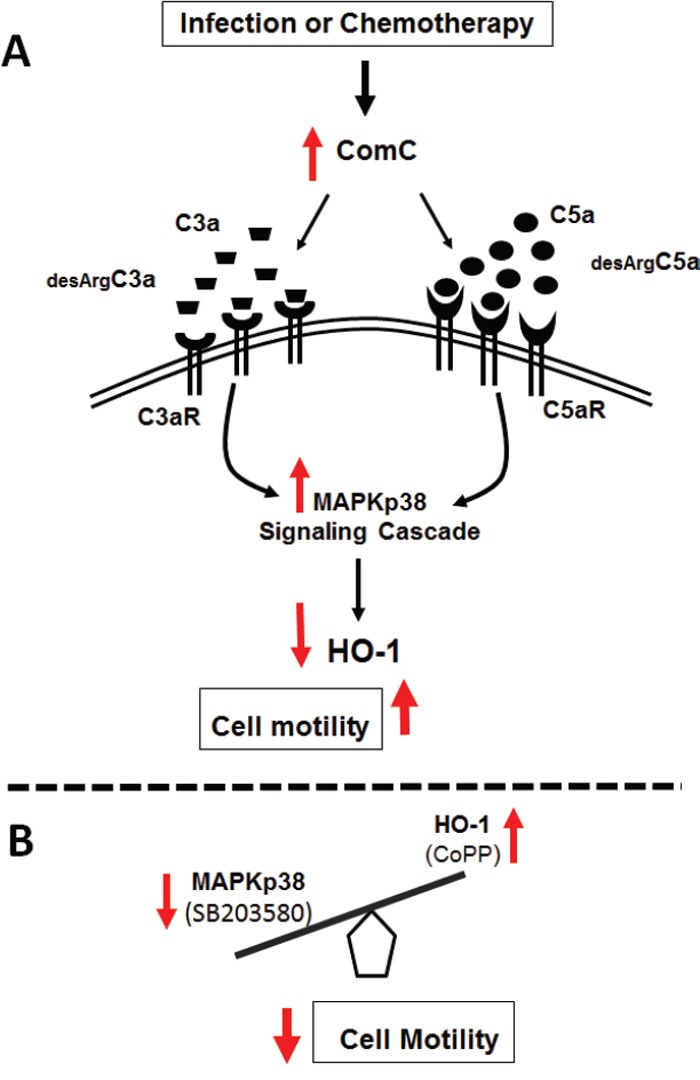
Molecular targets to inhibit the ComC-mediated increase in motility of leukemic cells - upregulation of HO-1 or inhibition of p38 MAPK Panel **A.** The pro-migratory effects of C3 or C5 cleavage fragments released during infection or chemotherapy lead to p38 MAPK-mediated HO-1 downregulation via their specific receptors. Due to these changes, leukemia cells become more mobile, resulting in spread of the malignant cells within hematopoietic tissues. Panel **B.** Based on our results, activation of the ComC-triggered migratory response of leukemia cells could be constrained using small molecules such as SB203580 and CoPP, which inhibit p38 MAPK and upregulate HO-1, respectively. [Modified from ref. 1].

We previously proposed (Figure 1A) that activation of the ComC in BM is negatively regulated by heme oxygenase 1 (HO-1) [3,4] in a p38 MAPK-dependent manner [4] and that, by upregulating p38 MAPK, the ComC downregulates HO-1 [4-7], and we tested the potential involvement of the C3aR/C5aR–p38 MAPK–HO-1 signaling axis in these phenomena. HO-1 is an inducible enzyme that is upregulated in response to several oxidative stress stimuli, and the anti-inflammatory functions of HO-1 have been very well demonstrated in HO-1-deficient mice as well as in human HO-1 deficiency, in which the ComC becomes hyperactivated due to the lack of a balancing inhibitory effect by HO-1 [8]. One of the anti-inflammatory effects of HO-1 is inhibition of cell migration [4-7]. Providing a mechanism for this inhibition, we have also recently demonstrated that upregulation of HO-1 in human leukemic cell lines decreases cell chemotaxis, while intracellular downregulation of HO-1 has the opposite effect [4].

In our experiments we found that stimulation of leukemic cells by C3 or C5 cleavage fragments activates p38 MAPK, which downregulates HO-1 expression, rendering the leukemic cells more mobile. Thus, this ComC-mediated pathway may enhance the unwanted spread of leukemic cells in the body. This phenomenon may affect leukemic cells clinically, as ComC activation often accompanies leukemia infection, or, more importantly, accompanies the spread of more resistant leukemic cells after unsuccessful chemotherapy. Based on the role of the C3aR/C5aR–p38 MAPK–HO-1 signaling axis in this unwanted phenomenon, we hypothesized that inhibition of p38 MAPK or upregulation of HO-1 by small-molecule modulators such as SB203580 and CoPP, respectively, could have a beneficial effect on ameliorating the expansion of leukemia/lymphoma cells during ComC activation (Figure 1B). Importantly, our in vitro studies, in which C3 and C5 cleavage fragments enhance the motility of malignant cells in a p38 MAPK–HO-1-dependent manner and may contribute to their spread, were subsequently confirmed in vivo in an immunodeficient mouse model using human leukemia cells exposed before injection to a small-molecule p38 MAPK inhibitor or HO-1 activator [1].

In conclusion, our results indicate that activation of the ComC in leukemia patients enhances the migratory potential of malignant blasts, and this enhanced motility may contribute to the systemic spread of leukemic cells [1]. We propose that small-molecule inhibitors of p38 MAPK or activators of HO-1 could ameliorate this unwanted effect.
